# First Responders to Hyperosmotic Stress in Murine Astrocytes: Connexin 43 Gap Junctions Are Subject to an Immediate Ultrastructural Reorganization

**DOI:** 10.3390/biology10121307

**Published:** 2021-12-09

**Authors:** Anja Beckmann, Johanna Recktenwald, Alice Ferdinand, Alexander Grißmer, Carola Meier

**Affiliations:** Department of Anatomy and Cell Biology, Saarland University, 66421 Homburg/Saar, Germany; Anja.Beckmann@uks.eu (A.B.); johanna.recktenwald@gmx.net (J.R.); s8alferd@teams.uni-saarland.de (A.F.); Alexander.Grissmer@uks.eu (A.G.)

**Keywords:** FRIL, Cx43, sucrose, hyperosmolar, freeze fracture, ultrastructure

## Abstract

**Simple Summary:**

Gap junctions are intercellular channels that provide the means for direct transport of small molecules, ions, and water between connected cells. With these functions, gap junctions are essential for the maintenance of astrocytic homeostasis and of particular importance in the context of pathophysiological disbalances. These include the hyperosmolar hyperglycemic syndrome or the pathology after brain trauma. We demonstrate that short-term hyperosmolarity reduces intercellular communication via gap junctions. These functional changes coincide with the transformation of gap junction ultrastructure as evidenced by freeze-fracture replica immunolabeling and transmission electron microscopy. The hyperosmolarity-induced immediate changes in the ultrastructural assembly of connexons, the protein constituents of gap junction channels, have not been described in astrocytes before and are revealing the coherence of structure and function in gap junctions. Phosphorylation of Connexin 43, the main gap junction protein in astrocytes, at amino acid 368 (Serine) might link the two.

**Abstract:**

In a short-term model of hyperosmotic stress, primary murine astrocytes were stimulated with a hyperosmolar sucrose solution for five minutes. Astrocytic gap junctions, which are mainly composed of Connexin (Cx) 43, displayed immediate ultrastructural changes, demonstrated by freeze–fracture replica immunogold labeling: their area, perimeter, and distance of intramembrane particles increased, whereas particle numbers per area decreased. Ultrastructural changes were, however, not accompanied by changes in Cx43 mRNA expression. In contrast, transcription of the gap junction regulator zonula occludens (ZO) protein 1 significantly increased, whereas its protein expression was unaffected. Phosphorylation of Serine (S) 368 of the Cx43 C–terminus has previously been associated with gap junction disassembly and reduction in gap junction communication. Hyperosmolar sucrose treatment led to enhanced phosphorylation of Cx43S368 and was accompanied by inhibition of gap junctional intercellular communication, demonstrated by a scrape loading-dye transfer assay. Taken together, Cx43 gap junctions are fast reacting elements in response to hyperosmolar challenges and can therefore be considered as one of the first responders to hyperosmolarity. In this process, phosphorylation of Cx43S368 was associated with disassembly of gap junctions and inhibition of their function. Thus, modulation of the gap junction assembly might represent a target in the treatment of brain edema or trauma.

## 1. Introduction

Gap junctional intercellular communication (GJIC) within the astroglial syncytium is the main component for brain homeostasis, for instance by enabling the spatial buffering of potassium [1]. Gap junctions are assembled of two connexons, with each connexon being composed of six connexins. Two connexons of adjacent cells form an intercellular gap junction channel and several of those channels assemble to a gap junction of variable size. Within the gap junction, cell membranes of the two connected cells converge to a distance of 2–3 nm, which is considered the structural equivalent for GJIC [2]. Besides their localization at astrocyte–astrocyte or at astrocyte–oligodendrocyte contact sites and in the vicinity of synaptic glomeruli [3,4], another important position of astrocytic gap junctions is in proximity to astrocyte end–feet and the blood–brain barrier, to the integrity of which they are likely to contribute [5,6]. Although Connexin (Cx) 26, Cx30, and Cx43 were shown to play a role in brain homeostasis, Cx43 is considered the main gap junction protein in astrocytes. A number of in vivo and in vitro studies, using pharmacological gap junction inhibition or the deletion or overexpression of Cx43, confirmed the relevance of Cx43 for astrocytic well-being [7,8,9].

Phosphorylation of Cx43 together with a short Cx43 half-life (1.5–5 h) is the opportunity of fast reacting modulation for the regulation of GJIC [10,11,12,13]. Within the amino acid sequence of Cx43 are 19 phosphorylation sites, specifically determining the assembly and turnover of gap junctions [14,15,16,17]. Phosphorylation regulates the binding of cytoskeletal proteins, for instance the zonula occludens (ZO) protein 1, and is thereby important for both the assembly and disassembly of gap junctions [18].

Recently, we demonstrated that metabolic inhibition of astrocytes through temporary oxygen and glucose deprivation with subsequent reoxygenation (OGD-R) led to a loosening of the gap junction particle assembly in combination with phosphorylation of Cx43 at its amino acid Serine (S) 368 [19]. To ascertain whether the observed changes in the ultrastructure of Cx43 conjointly with the phosphorylation at S368 are OGD–R–specific, or rather serve as a unique mechanism in the regulation of GJIC in astrocytes, we chose to treat astrocytes with a hyperosmolar sucrose solution (0.5 M) as was previously used by Peracchia et al. for induction of hypertonicity in liver [20]. We hypothesized that changes in osmolarity would affect both ultrastructure and function of astrocytic gap junctions. Indeed, hyperosmolar sucrose interrupted GJIC in cultured murine astrocytes as shown by a scrape loading dye transfer assay. We analyzed expression and phosphorylation status of Cx43 by immunoblotting, demonstrating an increase of Cx43S368 after sucrose treatment. This was accompanied by loosening of the gap junctional particle cluster as evidenced by freeze-fracture replica immunogold labeling (FRIL). Hyperosmolar sucrose resulted in even more pronounced effects on gap junction area and perimeter, as well as on the distance between nearest particle neighbors as compared to OGD–R treatment. In view of the crucial role of Cx43 in astrocytic potassium buffering, the observed changes might allude to early onset changes of hyperosmolarity affecting gap junction structure and coherence.

## 2. Materials and Methods

### 2.1. Culture of Primary Astrocytes and Determination of Microglia Concentration

Primary astrocytes were isolated from cerebral cortices of newborn C57BL/6–mice of either sex (postnatal day (P)1-5) as previously described [19] and cultured in growth medium (low glucose DMEM (Merck, Munich, Germany), complemented with 1% (*v*/*v*) Penicillin–Streptomycin (Thermo Fisher Scientific, Dreieich, Germany), 1% (*v*/*v*) non-essential amino acids (PAA laboratories, Coelbe, Germany), and 10% (*v*/*v*) fetal calf serum (FCS; Thermo Fisher Scientific, Dreieich, Germany)) for 3 weeks prior to use. In total, 18 astrocytic preparations were performed from independent litters. For each set of experiments, a minimum of three separate cultures was utilized. The average purity of cultures was 85.6% (±3.3 standard error of the mean (SEM)) as determined by the percentage of GFAP-immunopositive astrocytes (not shown) compared to the total number of cells. The remaining 14.4% of cells were identified as mostly CD68–immunopositive microglia/macrophages.

### 2.2. Induction of Osmotic Stress

Cells were seeded onto 12 mm diameter glass coverslips coated with 0.01% (*w*/*v*) poly–L–lysine (PLL, Merck, Darmstadt, Germany) in H_2_O (bidest.), which were placed in 24–well plates (Greiner Bio-one, Frickenhausen, Germany) for further cultivation and stimulation. For freeze–fracture analyses and immunofluorescence staining, 50,000 cells were seeded per coverslip. For isolation of RNA or protein, as well as for scrape loading dye transfer assays, 400,000 cells were seeded onto 35 mm diameter PLL-coated plastic culture dishes (Sarstedt, Nümbrecht, Germany). One day after plating, the growth medium was replaced by serum-reduced medium containing 0.5% (*v*/*v*) FCS.

Twenty–four hours later, the cells were incubated with 0.5 M sucrose solution (in PBS) at 37 °C for 5 min. The control groups were incubated with PBS solution. After that time, the PBS or sucrose solution were discarded and cells were processed depending on their further use: Cell layers on coverslips were fixed immediately with 500 µL ice–cold 2% (*w*/*v*) paraformaldehyde (PFA) in 0.15 M Soerensen’s phosphate buffer (SPB) for freeze-fracturing or with 99% ice cold ethanol for immunocytochemical stains. Cells for RNA or protein isolation were treated with either PBS or sucrose accordingly. After 5 min, the sucrose solution was replaced by 0.5 mL PBS. Adherent cells were abraded from the culture dish using a cell scraper, centrifuged, and stored at −80 °C until further processing.

### 2.3. RNA Isolation, cDNA Synthesis and Quantitative Real-Time PCR

The RNA isolation was performed using the Qiagen RNeasy Plus mini Kit (Qiagen, Hilden, Germany) according to the manufacturer’s instructions at room temperature. Removal of genomic DNA was ensured using a gDNA Eliminator spin column (Qiagen). After the RNA isolation step, an ethanol precipitation was performed to concentrate the RNA. The final determination of RNA concentration was performed spectrometrically using the Nano Drop ND-1000 (Thermo Fisher Scientific, Dreieich, Germany).

Reverse transcription of the RNA (500 ng per sample) in cDNA as well as the quantitative real–time (q)PCR was performed as described before [21]: Sequences of specific primers for Cx43, ZO–1, and 18S RNA are listed in Table 1. Samples of three independent experiments were measured in triplicate. For calculation of relative mRNA expression according to Pfaffl [22], the mean Ct of triplets from genes of interest was related to the mean Ct of 18S rRNA and the ratio 2^-ΔΔCt^ was calculated using Microsoft Excel.

### 2.4. Cell Lysis and Immunoblotting

Protein isolation was performed using radioimmunoprecipitation (RIPA) lysis buffer as described previously [19]. Fifteen µg of protein extracts were separated by SDS–PAGE. Immunoblotting (IB) was followed by incubation with milk powder solution (1% (*w*/*v*), diluted in TRIS–buffered saline (TBS) complemented with 0.05% (*v*/*v*) Tween20 (Carl Roth, Karlsruhe, Germany) (TBST) for 30 min. Primary antibody (Table 2) incubation was carried out at 4 °C overnight. After washing in TBST–solution, horse radish peroxidase–conjugated secondary antibodies goat anti–rabbit (Thermo Fisher Scientific, Dreieich, Germany), goat anti–mouse (SantaCruz Biotechnology, Heidelberg, Germany), and donkey anti–goat (Santa Cruz Biotechnology, Heidelberg, Germany) were all diluted 1:7000 in 1% milk powder solution (see above) and incubation was performed at room temperature for 1 h. Electrochemiluminescence detection was performed using WesternBright Quantum ECL substrate (Advansta, San Jose, CA, USA). Signal intensities were used for calculation of the relative protein expression using Microsoft Excel.

### 2.5. Immunocytochemical Staining

Coverslips had been fixed with 99% ethanol at −20 °C for 20 min and were subsequently air dried. Dried coverslips were stored on filter paper at −20 °C until further processing. Staining was performed as described previously [19] and included a blocking step in 10% (*v*/*v*) normal goat serum (NGS) and 0.1% (*v*/*v*) Triton X-100 in PBS. Afterwards, the coverslips were incubated for 2 h with primary antibodies (Table 2) followed by blocking in 0.2% bovine serum albumin in PBS (*w*/*v*) for 30 min. The corresponding secondary antibodies (Alexa–Fluor^®^488 goat anti–mouse, Alexa–Fluor^®^568 goat anti–mouse, Alexa–Fluor^®^488 goat anti–rabbit, and Alexa–Fluor^®^568 goat anti–rat; all from Thermo Fisher Scientific) were used in a 1:3000 dilution and cells were incubated for 60 min. Finally, coverslips were mounted onto microscope slides using DAPI–Fluoromount g (Southern Biotech, Birmingham, AL, USA). Analysis of Cx43 (abcam) and ZO–1 (Thermo Fisher Scientific, Dreieich, Germany) immunofluorescence was performed on images of PBS– (*n* = 2) and sucrose– (*n* = 3) treated cells. Using the square profile function of the software Zen lite 2012 blue edition (Carl Zeiss, Oberkochen, Germany), fluorescence maxima were determined on 3–5 square regions per image. Subsequently, the distance between fluorescence peak maxima was calculated according to Fischer et al. [25].

### 2.6. Scrape Loading/Dye Transfer Assay

For the scrape loading method [26,27], astrocytes cultivated on 35 mm cell culture dishes were treated with either 0.5 M sucrose solution or with PBS (control group) for 5 min. Preincubation with 50 µM carbenoxolone (CBX), a known inhibitor of gap junctions, for 30 min served as negative control. After a rinse with PBS, cells were incubated with a combination of Lucifer Yellow (LY, yellow fluorescence; 457 kD; 1 mg/mL) and Rhodamine-Dextran (RhD; red fluorescence; MW 10.000 kD; 10 mg/mL) dyes for 2 min, followed by scraping the cell layer in the center of the culture dish with a cannula (0.5 mm × 40 mm, B. Braun, Melsungen, Germany). After another two minutes, the cells were incubated in Ca^2+^- and Mg^2+^-enriched PBS in the dark for another 8 min. Dye-loaded cells were photographed and a minimum of three pictures was analyzed using the ImageJ software [28] according to the standardized procedure of Begandt et al. with slight modifications [29]. See Supplementary Figure S1 for further explanations.

### 2.7. Freeze Fracture and Immunolabeling

Cells from three independent astrocyte preparations were cultivated and treated with either PBS or sucrose as described above on coverslips and fixed in 500 µL 2% PFA in 0.15 M Soerensen’s phosphate buffer (pH 7.4). Vitrification using the cryo preparation chamber (Leica Microsystems, Wetzlar, Germany) as well as freeze-fracture and replication was performed as described previously [19]. Replication started with a 1 nm pre-carbon coat (applied with an angle of 90°), followed by a 1.5 nm platinum carbon coat (60°), completed by a second carbon coat of 20 nm (90°) at −160 °C and under vacuum conditions of 1.7 × 10^−7^ mbar. Replica stabilization [30] and immunolabeling was performed as described previously [19] using primary antibodies against Cx43 or P–Cx43S368 (Table 2) and secondary antibodies coupled to colloidal gold particles of 6, 12, or 18 nm sizes (all from Jackson ImmunoResearch, Newmarket, UK).

The labeled replicas were analyzed using a FEI Technai G2 transmission electron microscope (FEI, Hillsboro, OR, USA) at 100 kV. Pictures were taken with an 8-bit camera with an image size of 1.42 megapixel (Olympus, Hamburg, Germany). For each area of interest, two photos were taken with a goniometer angle of 8° between them, allowing stereoscopic analyses [31].

A maximum distance of 30 nm between gold and structure qualifies as specific labeling [32,33,34]. An average signal-to-noise-ratio (SNR) [31] of 1:2065 was determined for control replicas of PBS-treated astrocytes (*n* = 3), whereas the average SNR for replicas of sucrose-stimulated astrocytes (*n* = 3) was 1:1318. Both values are indicative of a highly specific labeling and a low background. The average labeling efficiency, which was determined by the number of intramembrane particles (IMPs) per gold, was 102 and 126 for replicas of control (*n* = 3) and sucrose-treated cells (*n* = 3), respectively. Examination of stereo pairs was performed to assess the location of gold beads with respect to the grid vs. tissue side of the replica. As the antibody targets a cytoplasmic epitope, the gold beads were located on the tissue side of the replica as expected [19,21]. Images were analyzed blinded using ImageJ to determine perimeter, area, IMPs per area, amount of gold particles, and the number of pits and particles. The center-to-center distances between nearest neighbors (nearest neighbor distance; NND) were calculated using the NND plugin from Mao [35]. For calculation of IMPs per area and the NND, only gap junctions formed by particles were analyzed.

### 2.8. Statistics

For each analysis, n-values and the exact p values are mentioned in the result section. Data are, if not otherwise mentioned, presented as mean plus SEM. First, a Kolmogorov–Smirnov test was performed as a normality test using GraphPad Prism 9.0 (GraphPad Software, San Diego, CA, USA). For further analyses of two groups, an unpaired *t*-test (for normally distributed data) or a Mann–Whitney test (for non-normally distributed data) was performed. As the FRIL data sets for the analyses of area, perimeter, IMPs per area, and the NND were non-normally distributed, a Mann–Whitney test with the test value *p* = 0.05 was performed. IB data were analyzed with an unpaired *t*-test for Cx43 total protein and ZO–1, and a Mann–Whitney test for phosphorylated Cx43. For analyses of qPCR data, Mann–Whitney test was used for relative RNA expression of Cx43. RNA expression of ZO–1 was analyzed with an unpaired *t*-test.

For analyses of multiple data sets of the SL/DT experiments a non-parametric test (Kruskal–Wallis test (Dunn’s multiple comparison test)) was performed for comparison of multiple groups. For the overall comparison of immunoblot results between groups, the Chi^2^ test was applied. For the comparison of individual immunoreactive bands, uncorrected Fisher’s LSD test was employed. Results of immunofluorescence maxima were analyzed using the Mann–Whitney test, as data were non-normally distributed.

Statistical significance was assumed with *p* < 0.05 and illustrated as follows (if not stated otherwise): * *p* < 0.05, ** *p* < 0.01, *** *p* < 0.001, and **** *p* < 0.0001, non–significant values (*p* ≥ 0.05) were not labeled. Histograms showing the relative frequency in percentages were compiled using GraphPad Prism 9.0.

## 3. Results

### 3.1. Influence of Short-Time Hyperosmolarity on the Ultrastructure of Cx43 Gap Junctions

Functional gap junctions are dynamic structures which display in freeze-fractured and replicated membranes as clustered pits (from an extraplasmic fracture face, the E–face) or particles (from a protoplasmic fracture face, the P–face), showing a characteristic periodicity in-between. The FRIL–technique [19,31,36] combines the identification of gap junctions by their morphology with the immunolabeling of their protein constituents using connexin-specific primary antibodies and gold-coupled secondary antibodies. In the electron microscopic images shown below, the black puncta are electron-dense immunogold puncta, indicating binding of antibodies. These are located within the gap junction area, labeled by green or blue overlays in control and sucrose-treated cells, respectively. Each of the pits (‘dimples’) or particles (‘bumps’) is the ultrastructural correlate of an individual connexon.

**Figure 1:**
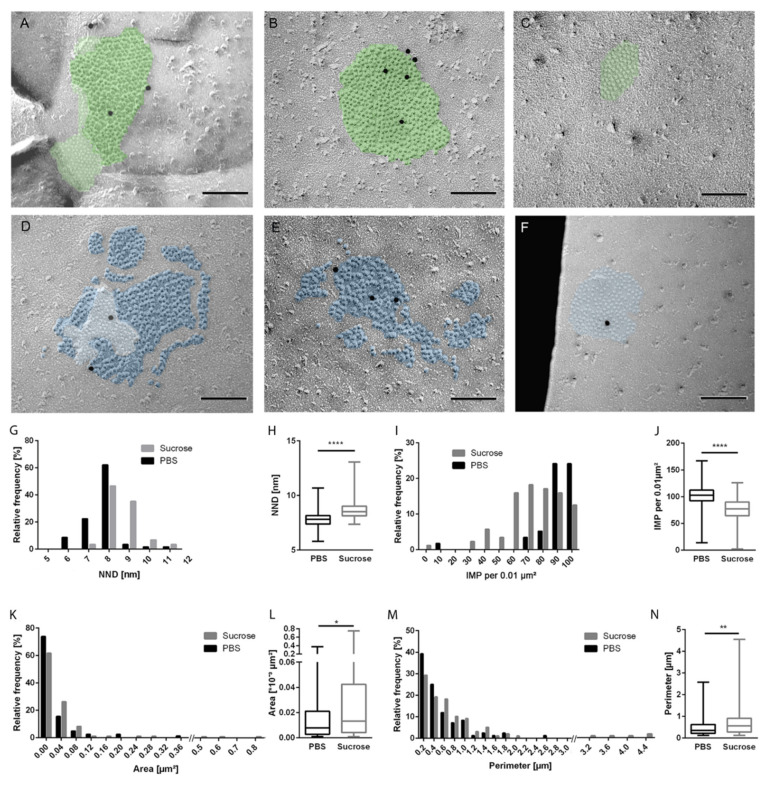
Sucrose loosened up the connexon arrangement within gap junction plaques. Astrocytic membranes were freeze-fractured and replicated after treatment with sucrose or incubation with PBS as a control. Replicas were labeled with primary antibodies against the C-terminus of Connexin (Cx) 43 and gold-conjugated secondary antibodies (black dots represent 12 nm gold). (**A**–**C**) Intramembrane particles (IMPs) in gap junctions of control cells appeared regularly arranged, and gap junctions featured both extraplasmic (E)– and protoplasmic (P)–faces (**A**) (pits are overlaid in light green and particles in darker green), P-faces only (**B**), or E-faces only (**C**). After sucrose treatment, the gap junction formation looked less compact and the IMP arrangement appeared dispersed on both E- and P-faces (**D**), pits with light blue overlay and particles in darker blue), only P-face (**E**) or only E-face (**F**). Quantification of freeze-fracture-replica immunolabeling (FRIL) data confirmed those observed changes. Histograms displaying the relative frequencies of (**G**) the distance between nearest neighbors, (**I**) the number of IMPs per area, (**K**) the gap junction area, as well as (**M**) the perimeter showed a clear shift upon sucrose treatment. Sucrose caused enlargement of the gap junction plaque without integration of new IMPs, as the number of IMPs per 0.01 µm² decreased, resulting in a shift to the left for sucrose-treated gap junctions (**I**). Those changes between gap junctions of control cells and sucrose-treated cells were significantly different (**H**,**J**,**L**,**N**). Statistical data were presented in box plots showing the median with whiskers for minimum and maximum. Statistic significances indicated in the figure are: * *p* <0.05, ** *p* <0.01, and **** *p* < 0.0001. Scale bars in (**A**–**F**) represent 100 nm. Adapted from [37].

We here labeled freeze-fractured astrocytic membranes with antibodies against Cx43. Gap junctions of astrocytes treated with PBS alone displayed the well-known arrangement of replicated connexons in E-face pits or P-face particles as shown in Figure 1A–C. After short-time hyperosmolarity, in contrast, most gap junctions looked less compact and the connexons appeared to be more distant from each other (Figure 1D–F). As we found all three forms of possible fracture faces of gap junctions, i.e., (I) gap junctions containing a fracture step between E- and P-face (Figure 1A,D), (II) gap junctions consistent of P–faces only (Figure 1B,E), or (III) gap junctions comprising E-faces only (Figure 1C,F), we reasoned that short-time hyperosmolarity did not generally cause the disconnection of gap junctions at cell-to-cell contact sides.

The loss of compactness observed within gap junctions after sucrose treatment was reflected by a loosened aggregation of particles within the gap junction. Therefore, we calculated the NND. Determination of the relative frequency of the calculated NNDs revealed a clear shift towards the right, i.e., to larger NNDs, after sucrose treatment (Figure 1G), reflecting smaller distances in control-derived gap junctions and larger distances between particles in sucrose-treated samples. In concordance, the median NND between intramembrane proteins (IMPs) also increased significantly (control 7.82 nm, 5.8–10.7 nm, *n* = 58/sucrose 8.5 nm, 7.4–13.1 nm, *n* = 88; *p* < 0.0001, Figure 1H). Thus, an expansion of particles was observed upon sucrose treatment with gap junctions displaying a 0.7 nm larger NND value than control cells. In addition to the analyses of NNDs, we counted and calculated the number of IMPs per area. Relative frequency analyses showed a shift to the left side after sucrose treatment (Figure 1I), pointing to lower numbers of IMPs per µm^2^. A significant decrease was detected in sucrose–treated samples (77 IMPs per 0.001 µm^2^ area; *p* < 0.0001) as compared to control samples (102 IMPs per 0.001 µm^2^ area; Figure 1J).

The loosening of gap junction compactness was not associated with a distinct enlargement of the average gap junction plaque, as measurement of the gap junctional area showed: Relative frequency analyses showed a shift to the right for gap junctions after sucrose treatment (Figure 1K), confirmed by statistical analyses of the average area (Figure 1L; control 0.0079 µm^2^, 0.001–0.3733 µm^2^, *n* = 84/sucrose treatment 0.013 µm^2^, 0.001–0.76 µm^2^; *n* = 103; *p* = 0.0208).

In accordance with the area, the perimeter of gap junctions was also affected. Relative frequency analyses showed a shift to the right side, i.e., towards increasing perimeter measurements, after sucrose treatment (Figure 1M) with the median being significantly different (Figure 1N; control 0.38 µm, 0.12–2.58 µm, *n* = 84; sucrose treatment 0.56 µm, 0.12–4.55 µm; *n* = 102; *p* = 0.0089). 

In summary, hyperosmolar sucrose treatment resulted in a significantly decreased number of connexons per area as compared to controls. This was reflected by an increased NND between gap junction particles together with reduced particle density, area, and perimeter in sucrose–treated astrocytes.

### 3.2. Influence of Short-Time Hyperosmolarity on the Gap Junction Protein Cx43 Expression

**Figure 2:**
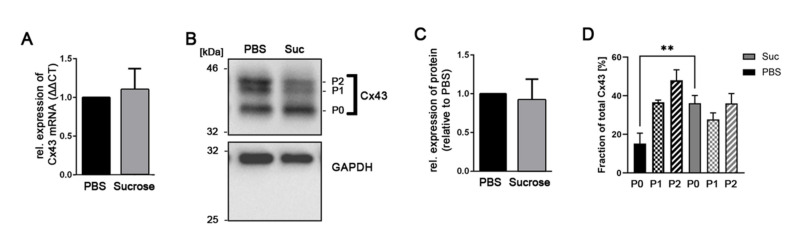
Sucrose does not affect Cx43 mRNA or protein expression. Astrocytes were treated with sucrose (*n* = 3) or with PBS as control (*n* = 3) for five minutes. (**A**) qPCR was performed with specific primers for Cx43 and results were normalized to 18S rRNA expression. Calculating the relative expression changes with respect to PBS-treated cells (=1) according to Pfaffl et al. revealed no changes. (**B**) SDS-PAGE of cell extracts and immunoblotting with an antibody recognizing all phosphorylation forms of Cx43 with GAPDH as a loading control were performed. Cx43 immunosignal showed unphosphorylated (P0) isoforms as well as two phosphorylated (P1/P2) isoforms. Original images are shown in Supplementary File S1. (**C**) Quantification of signal intensities, performed on the overall immunosignal per sample, did not show any significant changes of total Cx43 protein content. Shown are means + SEM of signal intensities relative to the control. (**D**) Differential quantification of immunopositive bands P0, P1, and P2 of PBS- (black columns) and sucrose- (gray columns) treated samples. Individual comparisons of P0, P1, and P2 band intensities between groups revealed significant differences for P0 bands (** indicates *p* < 0.0051; uncorrected Fisher’s LSD).

As FRIL analyses showed, sucrose treatment resulted in the loosening of Cx43 particle assembly within gap junctions. Analysis of Cx43 transcription by qPCR did not show a change in Cx43 mRNA expression (*n* = 3, 1.097-fold induction, *p* = 0.4, Figure 2A). 

At protein level, the typical pattern in Cx43-immunoblots are signals at the molecular weight of ~43 kDa, which represent the unphosphorylated (P0) form of Cx43, and one to three additional immunoreactive ‘bands’ (~46–48 kDa), which represent phosphorylated forms (P1 to P3, depending on the antibody used) [14,38]. In a first step, we analyzed the overall expression of Cx43, irrespective of its phosphorylation, by using an antibody directed against the C-terminal domain that recognizes unphosphorylated as well as phosphorylated Cx43 proteins. The total area of ECL-signal was determined in immunoblots and referred to the GAPDH signal for reference. Between both groups, the observed 0.9-fold change was not significantly different (*n* = 3, *p* = 0.8, Figure 2B,C). In contrast to the total Cx43 protein amount, the overall distribution pattern of the immunoreactive bands P0, P1, and P2 clearly changed between PBS- and sucrose-treated samples (Chi^2^ test; *p* = 0.003). Individual measurements of P0, P1, and P2 bands and their comparison between both groups (Figure 2D) revealed that the immunointensity of P0 changed significantly (uncorrected Fischer’s LSD (*n* = 3); PBS/P0 vs. sucrose/P0 *p* = 0.0051), whereas the immunosignal of phosphorylated forms did not (PBS/P1 vs. sucrose/P1 *p* = 0.1722; PBS/P2 vs. sucrose/P2 *p* = 0.0734).

### 3.3. Short-Time Hyperosmolarity Inhibits Gap Junction Communication

**Figure 3:**
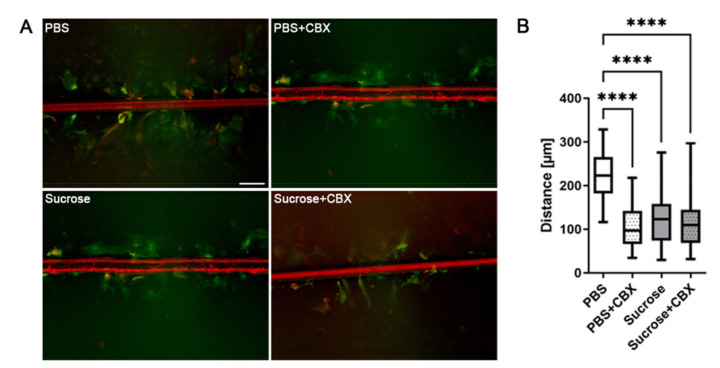
Sucrose inhibits gap junctional intercellular communication (GJIC) to the same extent as the gap junction inhibitor carbenoxolone. Functionally active gap junctions enable transmission of small molecules such as Lucifer Yellow (LY, shown in green) from one cell to neighboring cells, whereas high molecular dyes such as Rhodamine-Dextran (shown in red) are able to enter injured cells but remain there. (**A**) Cells incubated in PBS spread LY within the cellular network. This was reduced by the known gap junction inhibitor carbenoxolone (CBX). Sucrose treatment reduced the dye transfer to a similar extent as CBX, and this effect did not increase further by additional application of CBX (Sucrose + CBX). (**B**) Quantification of the distance of transferred dye (in µm) of *n* = 3 experiments are shown as box plots with median and whiskers for minimum and maximum. Statistic significances: **** *p* < 0.0001. Scale bar for all four images 100 µm.

As astrocytic gap junctions are an essential element of the glial syncytium in the intact brain, it is conceivable that pathological changes in osmolarity, mimicked by sucrose–induced hyperosmolarity in this study, might not only induce structural changes in gap junctions but also influence GJIC. To determine the amount of intercellular dye transfer as a measure for intercellular communication, a scrape loading-dye transfer assay was performed; photographs in Figure 3A are representative images taken from assayed cells. Cells under control conditions (treated with PBS) passed the Lucifer Yellow dye on to neighboring cells, reflected by the bright area in the images and indicative of functionally open gap junctions. Distances of dye transfer were measured (see Supplementary Figure S1) and quantified (Figure 3B): The dye passed a median distance of 222.5 µm (62 measurements). The well-established gap junction inhibitor carbenoxolone significantly reduced intercellular communication, resulting in a median distance of 96.75 µm (68 measurements, *p* < 0.0001).

Under hyperosmolar conditions, induced by sucrose incubation, dye transfer was significantly reduced: The scratched cells passed the Lucifer Yellow dye on in a range of 122.9 µm (median) only, which points to reduced GJIC. This result differs significantly from the results of the PBS control group (52 measurements, *p* < 0.0001, multiple comparison). Further inhibition via CBX did not result in an additional reduction in dye transfer (CBX: 59 measurements, 109.7 µm in median, *p* > 0.05).

### 3.4. Influence of Short-Time Hyperosmolarity on the Specific Phosphorylation of Cx43 at S368

**Figure 4:**
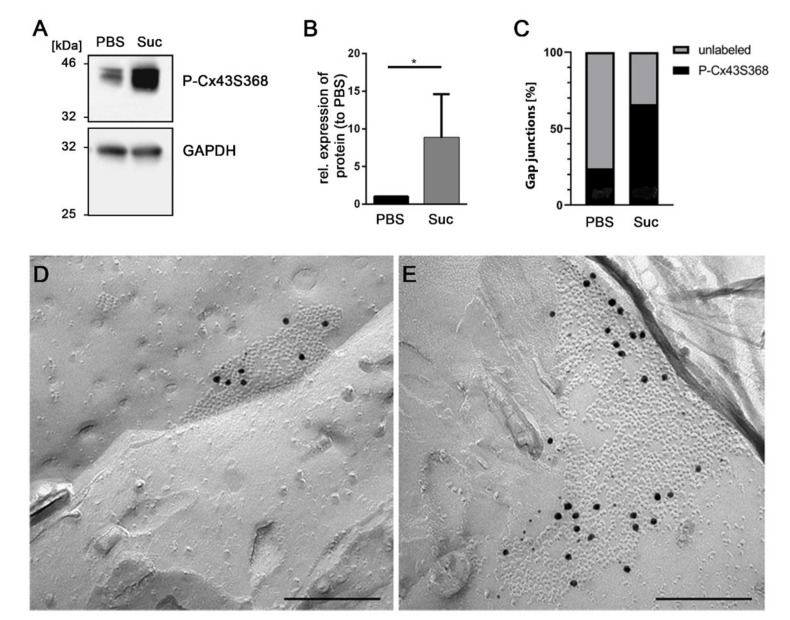
Sucrose-treatment results in phosphorylation of Cx43 at Serine 368. Antibodies recognizing the phosphorylation of S368 were used for Cx43-immunolabeling in sucrose-treated (hyperosmolar condition) or PBS-incubated (control condition) astrocytes in immunoblots and FRIL analysis. (**A**) The immunosignal of the Cx43S368 phosphorylated isoform was enhanced in sucrose-treated astrocytes (Suc) as compared to control astrocytes (PBS). GAPDH served as a loading control. Original images are shown in Supplementary File S1. (**B**) Quantification of *n* = 4 experiments confirmed a significant difference: * *p* < 0.05. Shown are signal intensities relative to the control (PBS), illustrated as mean + SEM. (**C**) Freeze-fractured replicas of control cells (PBS) or sucrose-stimulated cells (Sucrose) were labeled with antibodies recognizing the phosphorylation of S368. In replicas of *n* = 2 experiments, the number of gap junctions displaying phosphorylated Cx43S368-immunolabeling was determined. In comparison, 27 phospho (P)-Cx43S368-immunopositive gap junctions (black) and only 14 unlabeled gap junctions (gray) were found in membranes of sucrose-treated cells, whereas in membranes of control cells, only 7 gap junctions were P-Cx43S368-immunopositive (black) and 22 were found to be unlabeled (gray). (**D**,**E**) Examples of phospho-Cx43S368 immunolabeled gap junctions. Small and large black dots represent 6 nm and 18 nm colloidal gold, respectively, coupled to secondary antibodies. Although only one quarter of gap junctions of control astrocytes (PBS) displayed phosphorylated Cx43S368 labeled gap junctions, an image of one of these labeled gap junctions is shown (**D**). In contrast, 66% of gap junctions in sucrose-treated cells contained Cx43 phosphorylated at S368 (**E**). Scale bars represent 100 nm.

Although phosphorylation of Cx43 at Serine (S) 368 of its amino acid sequence is one of 19 phosphorylation sites known for Cx43, this particular phosphorylation site has previously been associated with reduced GJIC and gap junction internalization [10]. In addition to the analysis of overall Cx43 phosphorylation (see Figure 2), we specifically analyzed phosphorylation at S368. We applied an antibody, which specifically recognized phosphorylation at S368 of Cx43, in further immunoblot studies (Figure 4A). Analysis of signal intensities revealed an 8.8-fold increase of Cx43 phosphorylated at S368 (*n* = 4, *p* = 0.0286, Figure 4B).

Comparison of gap junctions derived from two replicas of either PBS– or sucrose–treated cells, labeled with specific antibodies recognizing phosphorylation of Cx43 at S368, also revealed an increase of the number of P-Cx43S368-labeled gap junctions upon sucrose treatment (Figure 4C). The ratio of P-Cx43S368-labeled gap junctions as compared to unlabeled gap junctions was significantly higher after osmotic stimulation with sucrose (ratio 1.94) as compared to PBS–treated cells (ratio 0.32) (Figure 4C). Exemplary images of sucrose-treated and untreated gap junctions labeled for phosphorylated S368 are shown in Figure 4D,E.

### 3.5. Influence of Short-Time Hyperosmolarity on Associated Proteins

**Figure 5:**
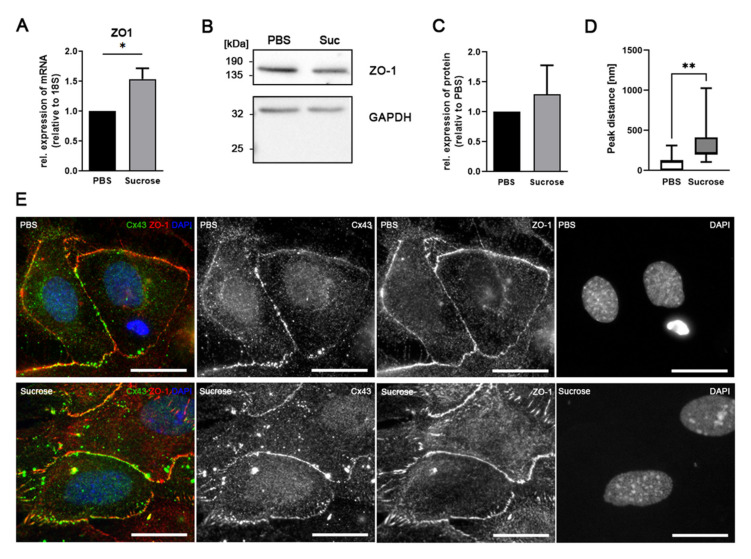
Hyperosmolar sucrose induces the transcription of zonula occludens (ZO) protein –1 at mRNA but not at protein level. (**A**) qPCR analyses of *n* = 3 experiments showed a significantly increased ZO–1 mRNA expression after sucrose-treatment (Suc) as compared to controls (PBS). Shown are means (+SEM). Statistical significance: * *p* < 0.05. (**B**) Immunoblotting with antibodies against ZO–1 showed no change in protein expression after sucrose-treatment. Immunolabeling with antibodies against GAPDH served as loading control. Original images are shown in Supplementary File S1. (**C**) Quantification of immunoblots (*n* = 4) confirmed the constant level of protein expression. (**D**) Quantification of immunofluorescent maxima in images of Cx43 and ZO–1 immunofluorescence. Whereas the fluorescence maxima at 488 nm and 568 nm excitation wave lengths were close-by in PBS-treated cultures, peaks were more distant in sucrose-treated cells, indicating less co-localization of the two detected proteins. Quantification of the peak distances (in nm) are shown as box plots with median and whiskers for minimum and maximum; ** indicate *p* = 0.0033). (**E**) The first panel on the left shows the merged immunofluorescence color images of Cx43 (green) and ZO–1 (red) proteins in PBS– and sucrose-treated astrocytes. Nuclei were labeled by DAPI (blue). Monochrome images show the immunofluorescence of Cx43 (second panel) and ZO–1 (third panel) as well as DAPI (fourth panel) separately. Although cell membranes and cellular contact sites are labeled by Cx43 and ZO–1 under both conditions, the distribution of the two immunosignals seems to change upon sucrose treatment. Scale bar: 20 µm.

The ZO–1 protein is not only an integral part of tight junctions, forming the diffusion barrier between cells, but is also interacting with the C-terminus of Cx43 and, by this, participating in the regulation of gap junction size [39]. Therefore, we analyzed mRNA and protein expression of ZO–1 upon treatment with hyperosmolar sucrose. mRNA level of ZO–1 increased significantly (*n* = 3, 1.5-fold increase, *p* = 0.039, unpaired *t*-test, Figure 5A) upon sucrose treatment, whereas ZO–1 protein expression did not change significantly (*n* = 4, *p* = 0.5699, Figure 5B,C). Co-localization analysis of Cx43 and ZO–1 immunocytochemistry revealed that the peaks of fluorescent maxima at 488 nm and 568 nm excitation wave lengths, respectively, were more distant in sucrose-treated samples (Figure 5D). Immunofluorescence images (Figure 5E) confirms this data in that both signals seem to diverge in sucrose-treated samples. At several contact sites, we could observe alternating immunosignals in PBS-treated cultures. In sucrose-treated samples, a punctate line of ZO–1 immunofluorescence (red) paralleled a line of Cx43 immunofluorescence (green).

## 4. Discussion

The gap junction protein Cx43 is most abundantly expressed in astrocytes and has an important role for astrocytic communication, which, in turn, is important homeostasis of extracellular ion (e.g., potassium) concentration and, in the case of increased levels of potassium, the maintenance of membrane isopotentiality [1,40,41].

Pathophysiologically, astrocytic Cx43 has been connected to ischemic stroke [42,43], to the formation of brain edema, for instance after ischemia or trauma [44,45], to cancers of diverse tissues (reviewed by [46]), or to the response to mechanical stress [47], making the regulation and functionality of Cx43 subject of many studies [48].

The short life cycle and fast turnover of connexins enable a fast adaption of cellular communication. Within these adaptation processes, protein modifications such as ubiquitylation and phosphorylation are well-studied mechanisms. Various phosphorylation sites, all of which are located within the C-terminal tail of Cx43, were associated with channel closing, connexin trafficking, incorporation of new channels, and assembly of gap junction plaques, as well as disassembly of gap junction plaques and connexon internalization [49].

In intact brain tissue, astrocytic gap junctions generally consist of the proteins Cx43, Cx26, and Cx30 [50]. As Cx43 is the only connexin protein expressed in cultured astrocytes [50], we chose to investigate the effects of hyperosmolarity on Cx43 in this study. We were able to show ultrastructurally that the morphology of Cx43 gap junctions was significantly affected even after a five-minute sucrose-treatment.

Connexons appear as particles in P-faces of freeze-fracture replicas. After sucrose stimulation, the number of IMPs per area decreased, whereas the gap junction area, coherent with their perimeter, clearly increased. Consistent with this finding, the overall distance between the IMPs, the so-called nearest-neighbor distance, increased as well. In total, these changes characterize the reduction in compactness within the gap junctional organization.

Due to the high resolution required, studies on channel assembly are rare. Two of these studies were performed in the liver, where Cx32 and Cx26 are the major connexin proteins present: Ultrastructural changes after altered homeostasis were demonstrated for Cx26 and Cx32 in hepatocytes of sucrose-perfused murine livers [36]. In contrast to the loosening of gap junctions demonstrated by Fujimoto et al., Perracchia et al. demonstrated a tightening of IMPs in gap junctions of liver and stomach after perfusion with hypertonic sucrose-EDTA solution [20] which, however, might be explained by the use of EDTA [51]. In a previous study, we reported ultrastructural changes of the gap junction assembly upon oxygen–glucose deprivation in astrocytes, a model mimicking the early phase of hypoxia [19]. Thus, changes in channel assembly appear to be a stimulus-independent mechanism in the regulation of GJIC and thereby of cellular homeostasis.

We did not observe significant changes in Cx43 mRNA or pan-Cx43 protein levels in sucrose-treated astrocytes. This might be due to the short period of sucrose-treatment, not being sufficiently long for transcriptional or translational changes to occur. Our FRIL-data, however, showed distinct structural changes following even a short exposure time to hyperosmolar conditions. Additionally, functionally, channels closed up and dye transfer was significantly reduced. It is known that Cx43 channel activity and assembly is regulated by protein modifications including phosphorylation. Phosphorylation occurs via kinases, which act consecutively and cascade-like, and results in specific functional and structural changes of the gap junction (reviewed by [10]).

Disassembly of gap junctions, for instance, is known to occur after phosphorylation of S368 in astrocytes after oxygen–glucose deprivation with subsequent reoxygenation [19], after cardiac ischemia [52], and after keratinocyte scratch wounds [53]. The current study complements these data, as hyperosmolarity was also shown to result in an increased phosphorylation of Cx43-S368 in astrocytes. This is mediated by protein kinase C (PKC) and was shown to elicit conformational changes, associated with partial closure of Cx43 hemichannels [54]. In the present study, Cx43-S368 phosphorylation significantly increased in sucrose-treated astrocytes and was also associated with changes in the plaque structure and reduction in channel permeability to small molecular weight dyes. In immunoblots, we detected an alteration in the overall ratio of non-phosphorylated and phosphorylated Cx43 proteins, and we observed an increase of P0 non-phosphorylated forms. However, levels of P1 and P2 phospho–Cx43 did not change upon sucrose-treatment. In this context, the specific phosphorylation of S368 was surprisingly intense, but fit to the functional and structural outcome we observed. In view of a total of 19 phosphorylation sites within the C-terminal region of Cx43, it was to be expected that specific phosphorylation at individual phosphorylation sites, here S368, could be detected by specific antibodies only. Thus, they would go unnoticed by detection with a pan–Cx43 antibody, recognizing all phosphorylated and unphosphorylated isoforms. This might explain why phosphorylation changes could not be detected with respect to quantification of P1 and P2 immunobands.

These tight regulatory pathways of gap junctional function are of tremendous importance for astrocyte physiology. Associated with the disassembly of gap junctions after sucrose treatment was the decline of GJIC as shown by a scrape loading dye transfer assay. This functional observation correlates with studies, in which glial cells were treated with hyperosmolar solutions of different sugars or sugar alcohols: Zvalova et al. showed a reduction in GJIC in hyperosmolar sorbitol-stimulated astrocytes [12] and Muto et al. demonstrated a reduction in GJIC in retinal Müller cells after cultivation in high glucose medium [55]. Although both studies demonstrate an inhibitory effect of hyperosmolar solutions on gap junctional function, they do not refer to associated changes in gap junction ultrastructure. In terms of the experimental design of this study, it would be desirable to dye-inject astrocytes in situ, combined with determination of the potassium clearance [56]. This approach would provide more detailed information on the link of hyperosmolarity and gap junction function [56]. Alternatively, patch clamp-based approaches could be used for electrophysiological recordings, possibly followed by the addition of gap junction-permeable tracers to visualize the cellular network [57].

Astrocytes themselves are known to play an important role in the regulation of brain homeostasis, and particularly mediate potassium spatial buffering [1]. An unbalanced homeostasis leads to cellular damage or even death, mainly due to the incapacity of single cells or tissues to counteract an initial disturbance via regulating systems [58]. In the context of osmotic disbalance, the cellular syncytium requires rapid action in the form of gap junction closure to prevent damage, which is essential for astrocyte well-being. Sucrose-induced gap junction channel closure is therefore a very rapid reaction to extracellular hyperosmolarity in order to prevent the loss of water, which would follow the increased osmotic pressure in the outer space induced by sucrose. Besides the short-term closure and structural reorganization, indicating of the initiation of adaptive processes in response to hyperosmolarity, the reversal of these processes could also occur in a very fast manner. This plasticity distinguishes the described gap junction changes from regulation at the transcriptional or translational level, known, for instance, for aquaporin channels, which develop slower but are longer lasting.

Our present data prefigure Cx43 as a component of the cellular reaction system enabling the initial intervention to osmotic stress. The ZO–1 protein is known to be involved in the re-organization, disassembly, and internalization of gap junctions [48], thus, its increased mRNA expression after sucrose treatment might indicate its involvement in the structural changes observed. However, immunoblot data did not show an increase in ZO–1 protein. The reason for this may be the short treatment time (i.e., 5 min), not allowing translational changes to occur. Using the abcam anti-ZO–1 antibody, the major immunoreactive band was between molecular weight marker bands of 135 and 190 kD, although ZO–1 is generally detected at a molecular weight of 220 kD. Cross-reactivity to other ZO-protein isoforms such as ZO–2 has not been reported for this antibody, and the overall sequence similarity of ZO–1 and ZO–2 proteins is only about 54% [59]. However, as a) the molecular weight of the ZO–2 protein is 160 kD and b) there is a high sequence similarity in a certain region of the two proteins (ZO–1 aa 420 to 860; ZO–2 aa 93 to 524) [60], the detection of ZO–2 cannot be excluded.

Despite unchanged ZO–1 and Cx43 protein expression levels in PBS- vs. sucrose-treated astrocytes, immunofluorescence data nevertheless point to their connection: The spatial distribution of the two proteins changed in response to hyperosmolarity. The observed ZO–1 and Cx43 co-localization at intercellular contact sites under PBS-conditions seemed to diverge upon sucrose treatment. Quantification was performed by analysis of the distances between fluorescence maxima at 488 nm and 568 nm excitation for Cx43 and ZO–1 immunofluorescence, respectively, and confirmed that the two signals diverged under hyperosmolar conditions. The observed redistribution is possibly due to disturbed ZO–1–Cx43–binding. It is known that the ZO–1–Cx43–binding is inhibited by the phosphorylation cascade of Cx43 at S373, S365, and S368 [13,61]. Subsequent spatial dissociation of Cx43 and ZO–1 immunosignal were also observed in astrocytes under OGD conditions, a treatment which also increased Cx43 phosphorylation at S368 [19]. As the time of sucrose treatment in this study was only 5 min, the changes are expectedly smaller than those observed upon OGD-treatment of astrocytes. While several studies suggest that the loss of ZO–1 in consequence of the above-mentioned phosphorylation cascade enables opening of single channels in order to enter the gap junction plaque [13,39], Thevenin et al. reported the internalization of gap junctions after individual channel closure upon ZO–1 release as an additional process [61]. In our study, sucrose-treatment resulted in a loosening of the gap junction assembly, which is characterized by an increase of gap junction size combined with a decrease of particle density and a loss of gap junction function. Thus, our data allow us to hypothesize a role for Cx43 in the first response to osmotic stress in that hyperosmolarity will result in phosphorylation of S368 combined with channel closure and breaking apart of single channels from the gap junction assembly. Our FRIL data applying the Cx43S368 antibody did not support the current model of a circular organization of gap junction plaques, in which aged and, after phosphorylation of S368, closed channels would reside in the plaque center, whereas newly docked and concurrently open channels will attach in the periphery of the plaque. The immunosignal for Cx43S368 was not only located near the plaque center but was spread all over the gap junctional area in our study. As in FRIL, only a gold bead-to-particle distance of <30 nm is considered particle-associated labeling [30,32], labeling at the margin of mediate to large size gap junctions represents peripheral labeling.

To sum up, the observed re-organization of Cx43 gap junctions, together with the demonstrated closing of gap junction channels and impaired GJIC, might be evidence of their role as first responders upon extracellular hyperosmolarity. This mechanism might provide a protection from the astrocytic bystander effect, preventing the forwarding of apoptotic signals associated with hyperosmolar events. In humans, these are, for instance, occurring in association with the hyperosmolar hyperglycemic syndrome (HHS) and are thereby quite frequent. Thus, the fast closure of gap junctions, for instance by the use of gap junction inhibitors such as carbenoxolone, might enhance the observed protective astrocytic effect even in a clinical context.

## 5. Conclusions

Short-term hyperosmolarity via sucrose reduces gap junctional intercellular communication in primary astrocytes. These functional changes concur with the immediate reorganization of gap junction morphology, resulting in dispersed connexons. Thus, sucrose modifies (a) gap junction function, (b) gap junction ultrastructure, i.e., connexon assembly within gap junctions, (c) connexin phosphorylation at Serine 368, and (d) binding of cytoskeletal proteins such as ZO–1. To induce these changes, a 5-min-exposure to hyperosmolar sucrose solution was sufficient, indicating that the effects on gap junction structure and function occur immediately. Thus, gap junctions can be regarded as first responders to hyperosmotic stress. These mechanisms might be relevant in a clinical context such as the pathophysiological mechanisms in hyperosmolar hyperglycemic syndrome.

## Figures and Tables

**Table 1:** Primer pairs used for quantitative real-time PCR.

Product	Sequence (5′–3′)	Product Size [bp]	Reference
18S RNA	fwd AAACGGCTACCACATCCAAG	155	[21]
	rev CCTCCAATGGATCCTCGTTA		
Cx43	fwd CTCCAAGGAGTTCCACCACT	123	[23]
	rev TGGAGTAGGCTTGGACCTTG		
ZO–1	fwd GAGATGTTTATGCGGACGGT	146	[24]
	rev AGCTGTTTCCTCCATTGCTG		

Forward (fwd) and reverse (rev) primer sequences are listed with their specific product sizes in base pairs (bp).

**Table 2:** Data of primary antibodies in alphabetical order.

Name	Species	Manufacturer	Order Number	Dilutions		
				ICC	IB	FRIL
CD68	rat	Abcam	ab53444	1:1000	-	-
Cx43	rabbit	Abcam	ab11370	1:100	1:6000	1:75
Cx43	mouse	Thermo Fisher Scientific	13–8300	-	-	1:50
GAPDH	mouse	Merck	MAB374	-	1:2000	-
GFAP	mouse	Merck	MAB360	1:100	-	-
panCx43	rabbit	Sigma	C6219	-	1:8000	-
P–Cx43S368	rabbit	LS Bio (Seattle, WA, USA)	LS–C380672	-	-	1:100
P–Cx43S368	rabbit	Thermo Fisher Scientific	48-3000	-	1:500	1:100
ZO–1	goat	Abcam	ab190085	-	1:1000	-
ZO–1	mouse	Thermo Fisher Scientific	33–99100	1:100	-	-

Overview of antibodies used in this study, including characteristics such as origin, order number, as well as dilutions employed in the different methods.—not used for this method; ICC immunocytochemistry; IB immunoblot; and FRIL freeze-fracture replica immunolabeling.

## Data Availability

The data presented in this study are available on reasonable request from the corresponding author.

## Supplementary Materials

The following information is available online at https://www.mdpi.com/article/10.3390/biology10121307/s1, Figure S1: Determination of dye-spreading by scrape-loading dye transfer (SL/DT). File S1: Original images of immunoblotting.
