# Supplementary material for: First Responders to Hyperosmotic Stress in Murine Astrocytes: Connexin 43 Gap Junctions Are Subject to an Immediate Ultrastructural Reorganization
Source: Biology (Basel). 2021 Dec 9;10(12):1307. doi: 10.3390/biology10121307 (PMC8698406; doi:10.3390/biology10121307)
Supplement: Supplementary file 1 [file biology-10-01307-s001.zip › biology-1449786 Figure S1 Determination of dye-spreading by scrape-loading dye transfer (SLDT).pdf]

Supplementary Material

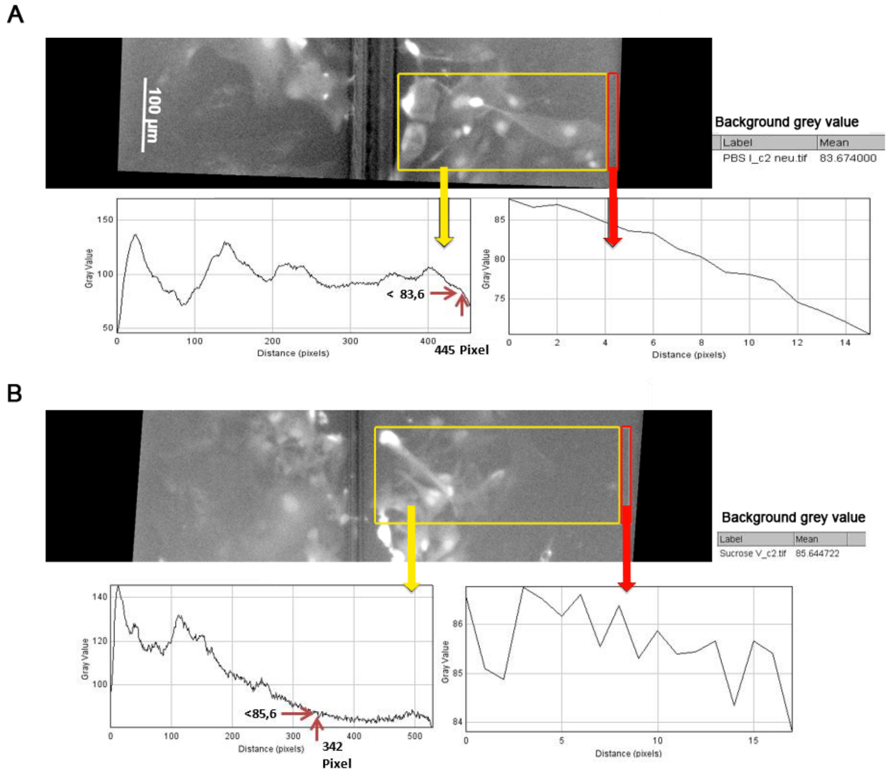

Figure S1: Determination of dye-spreading by scrape-loading dye transfer (SL/DT). Shown are exemplary grey scale pictures analysed for spreading of Lucifer yellow (LY) of PBS-control cells (A) and astrocytes after sucrose-treatment (B). A rectangle (as depicted in yellow) with a height of 200 pixels from the scratch to the background was drawn and the grey value of each pixel was determined using the plot profile function of Image J. Then, the mean grey value of the background (shown in red) was used to define the end of dye transfer distance. Appropriate plot profiles with indicated background values are depicted. The pixel values were converted to metric scale using Microsoft excel. Adapted from [36].
