# Supplementary material for: First Responders to Hyperosmotic Stress in Murine Astrocytes: Connexin 43 Gap Junctions Are Subject to an Immediate Ultrastructural Reorganization
Source: Biology (Basel). 2021 Dec 9;10(12):1307. doi: 10.3390/biology10121307 (PMC8698406; doi:10.3390/biology10121307)
Supplement: Supplementary file 1 [file biology-10-01307-s001.zip › biology-1449786_Supplementary File S1 Original images of immunoblotting.pdf]

Figure 2

3 4 9 10

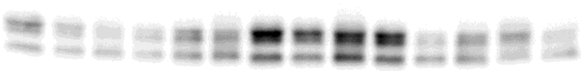

Cx43, shown are lanes 3 and 4, lanes 9 and 10 were used for quantification

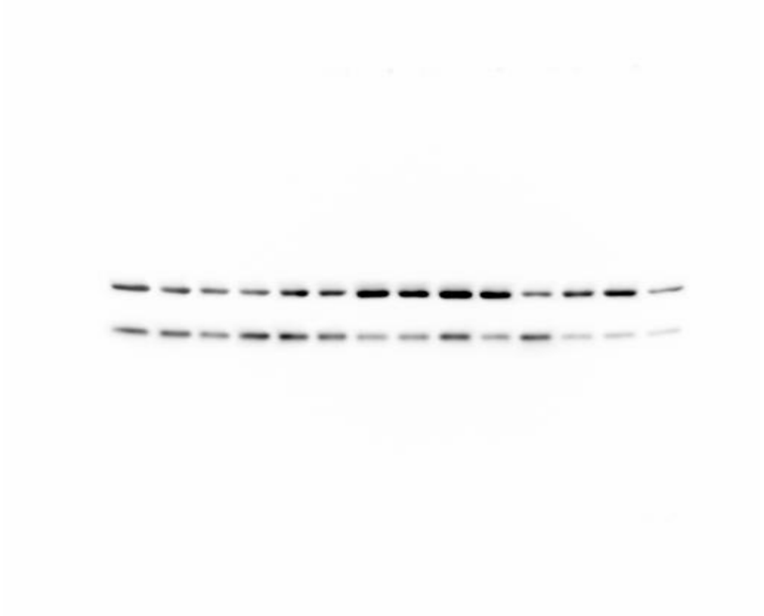

GAPDH (lower signal) for Cx43

Figure 4

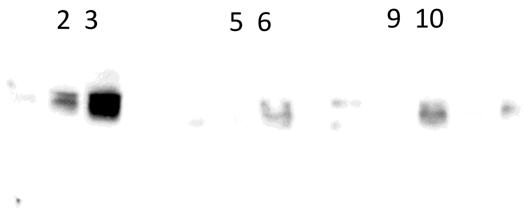

PhosphoCx43-S368, shown are lanes 2 and 3, lanes 5 and 6 and 9 and 10 were used for further quantification

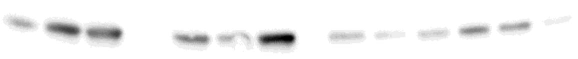

GAPDH for PhosphoCx43-S368

Figure 5

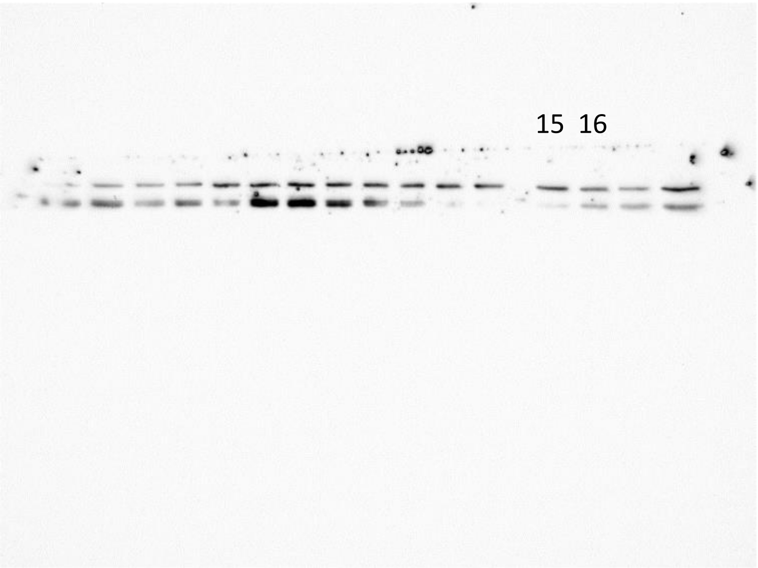

ZO-1 (upper signal), shown are lanes 15 and 16

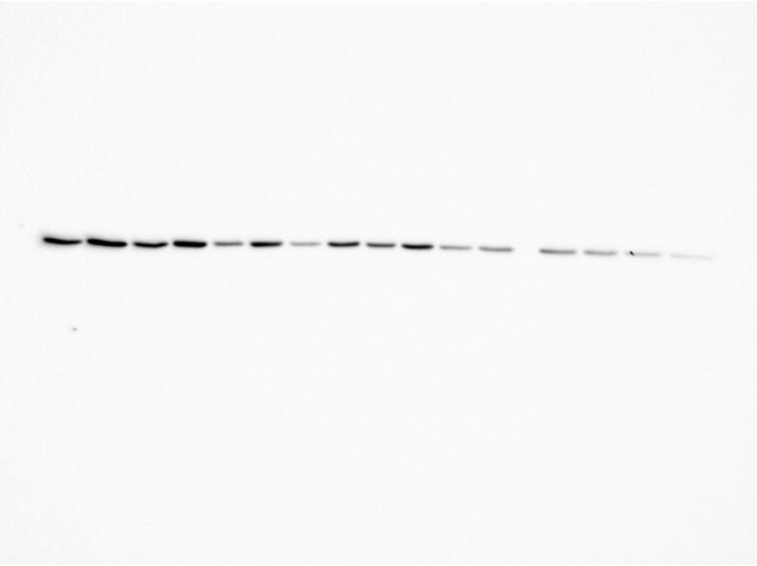

GAPDH for ZO-1
